# A general theory of consciousness II: *The language problem*

**DOI:** 10.1080/19420889.2022.2101194

**Published:** 2022-08-08

**Authors:** Abraham Peper

**Affiliations:** Department of Biomedical Engineering & Physics, Academic Medical Centre, University of Amsterdam, Amsterdam, The Netherlands

**Keywords:** Language, consciousness, cognition, adaptation, sensory, sensory images, inner speech, private speech, the self, visual thinking, verbal communication

## Abstract

It is generally assumed that what we hear in our head is what we think and that, when we tell a thought to somebody else, the other person understands what our thought has been. This paper analyzes how we think and what happens when we communicate our thoughts verbally to others and to ourselves. The assumption that we become conscious in language is erroneous: verbal communication is only an intermediary. The conscious experience of verbal communication is a sensory phenomenon. We think through sensory images (see Part I). This natural way of thinking, is a very refined and accurate method of translating thought into consciousness. It expresses our essentially unconscious neural cognitive activity in conscious sensory images: visual thinkers ‘see’ what they have thought. Why humans use verbal communication to express their thoughts to themselves is difficult to understand as the verbal way is extremely limited. The complex parallel cognitive activity has to be encoded into language tokens which are positioned sequentially as a string of symbols which somehow must express something comparable. Talking to oneself is directed to an imaginary person who is assumed to be the talking person himself. This imaginary person develops with the inner voice in infants and when the child grows up, that imaginary person remains there, somebody he talks to when he thinks and to which he attributes his feelings and his actions. The imaginary person is experienced as the human Self, but actually verbalizes the thoughts of the natural – animal – Self.

## 1. Introduction

Language has magical powers; you can enter somebody’s head and know what he thinks and you can let others know what you think and often you can let others think what you want them to think. These are some of the potentially positive properties of verbal communication, one of the most celebrated inventions in human history. This paper is about the negative aspects of verbal communication.

It is generally assumed that what we hear in our head is what we think and that, when we tell a thought to somebody else, that person understands what our thought has been. In this paper, I will try to analyze how we think and what happens when we try to communicate our thoughts verbally to others and to ourselves. In a previous paper on consciousness (referred to as Part I from hereon), I argued that to make us understand our neural cognitive activity, it is converted into sensory images which are fundamentally conscious [1]. There, the subject was the conscious experience of cognitive activity in general; here, the subject will be what happens when cognitive activity is translated into language – words – which will be shown to be essentially different. By means of the theory developed in Part I, I will demonstrate that much that is taken for granted about verbal communication is based on flawed premises and that the use of language is much less efficacious than is generally assumed [see also 2].

In Part I, I addressed four main issues: conscious new adaptive processes versus non-conscious automatic processes [see 1, 3, 4]; what consciousness is and how it is accomplished; how the different functions engaged in the consciousness process may be related and the translation of thoughts into consciousness. I argued that, in animals and humans, consciousness via sense organs is fundamental for the interaction they maintain with their environment while, in humans, consciousness is also achieved through spoken language. The present paper will consider different aspects of verbal communication, analyze verbal communication itself, discuss covert verbal communication and examine how the human Self evolves in verbal communication.

## 2. Consciousness and verbal communication

In the theory developed in Part I, thoughts – neural cognitive activity – become conscious by their expression in sensory images. There is an ongoing discussion about the nature and functioning of consciousness and particularly about what consciousness really is. But whatever consciousness is, or however it comes about, there is no doubt that it is an essential part of how the environment is perceived by the subject. As such, the senses and consciousness are inseparably bound. Sensory images are both the source of cognitive activity and the means of making the cognitive output conscious: the essentially unconscious neural cognitive activity is expressed in conscious sensory images [5, 1, 6]. This mechanism is evident in visual thinkers: they ‘see’ what they have thought .^a^

The translation of thoughts into sensory images is how living organisms become conscious of their cognitive processes. That sensory images are conscious, an experience known to all, is the basis of conscious thinking. Whether consciousness itself can be understood or is fundamentally illusive is not relevant here. For sensory information to become conscious, the role of cognition is essential: if cognition is not involved, we will not experience consciousness and we say that the process is automatic. But in automatic processes too, cognition is in a way decisive as it has shaped the processes in their adapting phase [1] and consequently determines process behaviour in recent automatic processes as well as in processes established long ago. That the involvement of cognition determines whether or not consciousness is experienced is addressed in Part I.

In humans, the natural sensory way of experiencing the outside world is supplemented and often dominated by the use of spoken language: instead of transforming thoughts about the outside world back to the sensory experience of the outside world, humans transform thoughts into verbal communication (see Section 5). Like all animals, humans perceive the real world in the natural sensory way, but the natural way is often not perceived as completely conscious. For animals, consciousness is in essence the awareness of the environment as expressed in sensory images. For humans, there is a distinct difference between their conscious sensory perception of the environment and the way the environment is experienced verbally. This difference is not due to sense experiences in humans in themselves being less conscious or non-conscious; it is a consequence of the way humans function in a world defined by language. For humans, verbal communication has become so dominant that sensory consciousness is often not experienced as genuinely conscious when it is not accompanied by covert or overt spoken language. An animal might look at a tree and experience it consciously. For a human, a tree usually becomes completely conscious only if a thought about the tree – or rather, a thought about the experience of seeing the tree – is put into words. The verbally expressed thought can then be communicated to others, which is a major goal in human life. For humans, just seeing a tree often does not meet the requirements of their world, which is based on interpersonal communication.

In most people, the natural sensory way of becoming conscious is obscured by verbal communication, although parts of it still can be dominant. Interoceptive sensations such as our feelings, for instance, play an important role in human life, although it is not clear whether it is as complex as in those who think completely sensorially [see 1, Section 7.4]. In those ‘visual thinkers’, the natural way is still the dominant way of becoming conscious of the world and of themselves. In preverbal infants, it is the way they think: it is innately present as it is in all animals.

A conscious experience perceived through the senses cannot be communicated to others; it is completely personal. In verbal communication humans therefore refer to other people’s conscious sensory experiences from the past. When I tell somebody that it smells of autumn outside, I do not convey the conscious sensory experience of the smell of autumn I had myself, but I refer to that person’s past sensory experiences of autumn smells. In spoken language, this is the closest we can come to communicating sensory experiences. But experiences always differ across individuals and it is consequently not possible to communicate my experience of the smell of autumn in an exact way to others [see e.g. 7].

Consciousness is part of the sensory mechanism; language, or rather the use of words, is in itself not conscious. The words are symbols referring to sensory images, which are conscious [see e.g. 8]. The sound of words is of course heard consciously, but the sound is only an intermediary, a tool to excite the sensory images constituting the verbal message. Hence, verbal consciousness, i.e. consciousness evoked by the words used in talking (see Section 5), is essentially sensory consciousness. The conscious experience that verbal communication evokes is a sensory phenomenon, similar to the natural conscious experience .^b^ This subject is treated extensively in the theory of grounded cognition [9], [10] 11–16]. Referring to conscious sensory images in the listener is the way conscious thoughts are communicated verbally. It constitutes the brilliant trick verbal communication is based on.

In summary, when we talk – to others or to ourselves – we express our thoughts in language, or rather, because we talk, in verbal communication. Telling our thoughts to ourselves we call thinking in language or verbal thinking. In reality, however, we become conscious of those thoughts through sensory images referred to by language. This is a confusing situation, all the more so because of the strong impression we have that we are conscious of our thoughts in language. But apparently, language is only an intermediary in the process of becoming sensorially conscious of our thoughts and the real problem then is that those thoughts cannot be expressed accurately through language.

## 3. The limitations of verbal communication

The cognitive system and the sensory system are extremely complex [see e.g. 17]. But as these structures must have developed concurrently, the conversion of the neural cognitive output into conscious sensory images can be assumed to be a fully adequate process. Thinking through sensory images is a very refined and accurate method of translating thought into consciousness. All of the senses are available to achieve a sensory image that expresses the outcome of the cognitive process accurately, while it is extremely fast.^c^ Visual images, the most prominent of sensorial images, are two- and often three-dimensional and can contain countless detail and meaning while the spatial composition in itself provides meaning and nuance.

Transforming the output of the neural cognitive process into verbal communication, however, poses major problems. Cognition has to express its complex activity in the restricted medium of language, which lacks the unique descriptive quality of the natural process [1]. Spoken language is one-dimensional and time-consuming: words are placed in a time sequence to form a composition which must make the subject aware of the outcome of his complex cognitive process. The transformation of thoughts into verbal communication is a process that differs fundamentally from the natural process of transformation into conscious sensory images. It is an artificial manipulation of a thought into a form that can be used for communication and the outcome does not reflect the thought very well. Verbal communication offers a poor representation of the thought it tries to express and it does not accurately reflects the essence of the cognitive output.

In humans, there apparently exist two conscious sensory versions of a neural thought side by side: the natural version and the artificial verbal version. The presence of these very different ways of expressing our thoughts is known to everybody. It is generally experienced as what we ‘know’ – through language – and what we ‘feel’. But these two seemingly different experiences are essentially representations of the same neural cognitive process: the natural sensory version – of which feeling is a part [1] – providing the natural transformation of the thought into consciousness and the simplified and curtailed version created via the verbal route.

The fact that the cognitive process is much more complex than language is able to express exposes a serious difficulty in verbal communication. A complex parallel cognitive activity has to be encoded into language tokens which are positioned sequentially: a complex thought is converted into a string of symbols which somehow must express something comparable. It is impossible for the huge complexity characterizing the neural mechanism of thinking to be expressed faultlessly in the limited design of spoken language [see 18, 19] and any transformation into spoken language must therefore lack accuracy.^d^

Apart from a difference in complexity, there is also a difference in structure hampering the transformation of neural thought processes into spoken language. An exact translation of a text from one spoken language into another is not possible, as all translators know, and an acceptable result is often difficult to achieve. A translation from a completely different medium, unrelated to spoken language, therefore seems an extremely difficult task and the result can never be more than a rough approximation.

A major problem is the actual way the process of verbal communication takes place. As indicated above, a visual thinker thinks in the natural way, just as all animals do: his thoughts are directly translated into sensory images. Unlike what ‘visual thinker’ suggests, however, a visual thinker does not think solely in visual images. In most cases, a thought is translated into a complex of different sensory images [see also 15, 20]. Part I cites Einstein, a visual thinker: *The psychical entities which seem to serve as elements of thought are certain signs and more or less clear images which can be “voluntarily” reproduced and combined. The above mentioned elements are, in my case, of visual and some muscular type*.^e^ This is what was conscious to Einstein. Generally, a conscious image is composed of images of many senses, including interoceptive sensations, but it can be expected to differ across individuals. When such a complex thought is then communicated verbally to another person, its various components have to be summarized and verbalized, which makes the translation process discussed above even more difficult.

In addition to the accuracy problems afflicting the translation of neural thought processes into spoken language, there is the difficulty that spoken language as a method of communication is itself fundamentally ambiguous [21–26] and it is of course not possible to translate an exact proposition accurately into an ambiguous medium. This problem is a serious one affecting form as well as content. Sentences, statements and whole manuscripts are ambiguous, as is demonstrated by for instance the voluminous exegesis of works by the various philosophers. The difference between what I think I say and what others think I mean can be enormous and the practical consequences for human societies are immense, existing in all probability from the birth of spoken language. This subject will be discussed extensively in Part III.

## 4. Practical implications

The problematic nature of the translation of thoughts into language is a highly fundamental and serious problem humans face when they want to communicate their thoughts verbally to others. But even with a theoretical language which is not ambiguous and evokes in the listener the exact meanings of the words used, the main purpose of verbal communication cannot be realized^f^. In essence, when I use verbal communication, it is my intention to evoke in my listener a thought I had, or to evoke a thought in the listener I want him to have, which ultimately amounts to more or less the same thing. This is practically impossible, however. As pointed out above and in Part I, my thoughts are complex neural processes which lack a well-defined relation with what the medium of spoken language can convey. When I translate a complex thought into spoken language, something potentially very different from my original thought results. This translation process is personal and cannot be known to others, and – an highly consequential fact – it cannot even be known to myself. All I know in language about the thoughts I have is the outcome of the translation into spoken language. In many people, there is some knowledge about the thought through the natural conversion process, but in most this is not clear and often restricted to the interoceptive component: a more or less vague feeling about the meaning of what they want to say.

What I say apparently differs from my thoughts. This is generally known. It is often difficult to express thoughts to somebody else and several endeavours are then necessary to achieve a satisfactory result. At the same time, people feel that they know what their thoughts are, which implies that the natural transformation into consciousness is in one form or another present in the background. My listener, however, does not have this information and the thoughts he arrives at are derived from the words I use, which he assumes to represent my thoughts. In short, I generally do not know exactly what my thoughts have been when I tell somebody what I assume is what I think, and the listener has only my words, which can be far removed from my thoughts.

The imperfections of verbal communication referred to above are alleviated by the participants sharing the context of the communicated information. However, any knowledge about the context suffers from the same difficulties as the individual words establishing it so that participants might well perceive that context differently. In simple language use, there are few problems. In a conversation about football, the problem that *the goal* and *a goal* can have essentially different meanings does not lead to much misunderstanding. But in complex and complicated matters, like politics, the context can be very much misunderstood.

It is generally taken for granted that the accuracy of verbal communication is appropriate in most circumstances. It should now be clear that verbal communication is a flawed and restricted method of exchanging thoughts and that the outcome of conversations may involve a high degree of misunderstanding.

## 5. The Self and the voice in my head

When a child is learning to speak, a peculiar phenomenon develops: the verbal Self. This human Self is fundamentally different from the animal Self. An animal perceives the outer world through its senses, contemplates these experiences and then reacts, either consciously or automatically. The Self of the animal is the organism as it functions and as it experiences the world through its senses. The Self is the realization of the animal’s position in its environment, its place in the group it belongs to, and the interaction of the animal with the others. The Self is what the animal thinks about, its situation and the actions it takes when it decides to act [see e.g. 27, 28]. Or to put it more succinctly, the Self of an animal is what the animal is: the Self of a duck is the duck itself.

Much has been said about the human Self [see e.g. 29]. The human Self is completely different from the animal Self as it is strongly associated with the inner voice. That humans talk to themselves is one of the most remarkable aspects of the use of language. It is so common that nobody experiences it as being strange; it is apparently seen as what humans do. But talking is communication and when humans turn their thoughts into spoken language, this is necessarily part of a communication process. And as communication is fundamentally between a sender and a receiver, there has to be somebody who listens when a person talks to himself.

The Self as we know it develops in children when they learn to speak. When a child starts talking to itself during that learning process, it communicates with an imaginary person, somebody it makes up, but who really feels alive [see also 30–32] much like the child imagines the teddy bear it plays with and talks to being alive. And when the child has grown up, that imaginary person is still there, somebody the adult talks to when he thinks and to which he also attributes his feelings and his actions.

Covert verbal communication – verbal thinking, the voice in my head – is directed to a fantasized person who at the same time is assumed to be the talking person himself. That imaginary person obviously does not exists, it is a creation. This is why the Self is so difficult to define: the Self of a talking human does not exist as a defined entity. It is a fabrication by the individual, originating in his childhood, and it is consequently completely personal.

The Self is an imaginary person who seems to look, feel and experience and communicates that in language. But those feelings and experiences are those of the person himself, his animal Self. This entails a curious fact: the whole procedure of turning thoughts into language and then telling myself – the voice in my head that tells me what I have thought – does not yield extra information. On the contrary, as is clear from the above, the sensory information from the language procedure is a much poorer representation of my thought than the natural version.

## 6. Verbal thinking

It is generally assumed that verbal thinking – covertly telling myself what my thoughts have been – is a way of thinking completely different from the animal way of thinking. However, as observed above, the assumption that we become conscious in language is erroneous and is probably caused by the strong auditory impression the accompanying speech evokes during verbal communication. We think through sensory images, just like animals do. That we also use language to communicate our thoughts to others is indeed an important difference with animals, but that humans use verbal communication as a way to express their thoughts to themselves is peculiar.

Verbal communication is useful for exchanging information between people despite the many serious drawbacks highlighted above. But that humans also use verbal communication to become conscious of their own thoughts makes no sense as the natural way is near perfect while the verbal approach is extremely limited and troublesome and burdens its users with many unnecessary problems. The assumption that verbal thinking is the perfect way of thinking and that it is the origin of all great accomplishments by the human race is puzzling. Talking to yourself, overtly or covertly, has no function as it does not tell you anything which cannot be learnt in the much more accurate natural way, while it greatly muddles the thought process. That there is no need for verbal communication in thought is demonstrated convincingly by visual thinkers, who fundamentally think without language.

The extent to which humans think in the natural sensorial way varies widely. It is a spectrum, ranging from the extreme, as in Einstein, to mere forms of feeling in people who think chiefly verbally [33]. Whether somebody may be called a visual thinker is therefore arbitrary. But in most people, natural thought is not very conscious. However, in mainly verbal thinkers, visual processes are active too as is demonstrated by 34.

The ‘visual’ in visual thinking is a simplification because all senses participate. But in animals with sight, the visual experience is generally dominant and that is what is most notable in ‘visual thinkers’. Most visual thinkers are not as extremely positioned as Einstein was and they all combine the visual thought process with verbal communication. In most cases, they interact verbally at the same level as verbal thinkers. The problems associated with translating thoughts into language do not differ much in visual thinkers. Like verbal thinkers, they have to translate a neural cognitive process into spoken language. The fact that they are also visually aware of the neural thought, while language thinkers are not, makes no difference as far as the translation problem is concerned.

## 7. The effect of language use on thinking

Above, I have looked at the problem of how the neural mechanism of cognition must try to cope with the restricted possibilities of verbal communication and I have analyzed the problems humans face when they communicate with others and with themselves. The problematic nature of the verbal communication process is worrisome as it hinders the thought process itself. However, reality is worse. In all probability, the cognitive process in verbal thinkers anticipates the problems presented by the continuous use of language and has adapted to the limited possibilities language offers [5,8].

While it is not likely that language use affects the neural process of thinking directly, the output of the cognitive process in humans is generally in language, which means that the analysis of problems will often be restricted to solutions afforded by language. This is a major complication. In addition, as verbal communication is a social event by definition, human thought is largely confined to what the social environment offers [see e.g. 7], narrowing the thought process even further. What has remained conscious of the extensive system governing human sensory thinking are emotions and feelings, which, however, cannot be put into words in any accurate way – perhaps explaining why it still exists in verbal thinkers. The meaning of the feeling component therefore always remains vague. In addition, those feelings often also originate from language use, making their value dubious, given the considerations above.

The natural way of thinking – problems are solved in a way that is optimal for a given animal, and made conscious via a translation into sensory images – is replaced in humans by verbal thinking, which largely limits and distorts the thought process. Visual thinkers do not show this effect; they think in the optimal – natural – way developed by humans through their evolution. Although they are theoretically able to compare the verbal output with the sensory output, correcting the verbal output for any inaccuracies will yield the same problems as in verbal thinkers. Consequently, in practice, there is unlikely to be much difference in the accuracy of verbal communication between verbal and visual thinkers.

The general attitude that verbal thinking is a positive factor in life is apparently unfounded. The natural way of thinking is optimal while the restricted possibilities of language constitute a serious restriction on the human thought process and the social interference component in the verbal adaptation process is an important cause of misunderstanding between groups of humans. This situation is a serious one as it not only plays a role in domestic social communication but is clearly paramount in politics

## 8. Discussion

Communication is a natural occurrence in nature, even down to cell level [35–37]. But there is a large difference between the innate communication in living organisms, where the language is fundamentally fixed and provides for an accurate and efficient communication process, and human communication, which is a more or less loose arrangement between participants with the message never completely clear. Having said that, the development of human verbal communication has of course been a tremendous and far-reaching achievement. As such, it has been celebrated through the ages and its use is still increasing due to the continuous growth in digital communication. But that there are fundamental negative aspects to the use of verbal communication is usually not realized. When a statement is not perceived or understood as was intended, the speaker generally attributes the miscommunication to an assumed lack of intelligence or dubious intentions on the part of the listener.

The present paper has tried to point out the inherent limitations of the use of language and the impossibility of communicating a thought accurately. Verbal communication is a useful tool when used in non-critical circumstances. It is a fundamentally flawed tool when its accuracy is of real importance, as in politics or science. Why verbal communication has become the human way of thinking in stead of just a tool for communicating messages to others is rather mysterious as the many limitations and inaccuracies of language are a daily experience for everyone.

What actually happens when I think verbally is that I tell my Self in words what my thoughts are. The fundamental question then is why I want to know what I think in words when I can learn that in the much more accurate sensory way. There is no satisfying answer. It is the human aberration. Humans function through verbal communication: everything they experience has to be told to others and when there is nobody to listen, they fantasize somebody. And in the end, they fantasize themselves as somebody else they can talk to. But its verbal adventure has left the human race with a poor understanding of its own thoughts as the ability to understand its thoughts in the accurate natural way is largely lost.

A solution to the problems set out in this paper might be to develop ways to prevent children from making the switch to verbal thinking when they are in the process of learning language. This would preserve the children’s inborn visual thinking capability while they still develop the normal language skills. Verbal communication could then become what it should have remained all along: a tool for communicating thoughts to others only.

## Notes

a Visual thinkers use visual language to express their thoughts. However, the term visual language is often also used to indicate a translation of verbal language into visual symbols (like the use of emoji). This is an unfortunate situation as the use of visual symbols in a text is not related to the way visual thinkers become conscious of their thoughts [38]. proposed denominating the verbal use of visual symbols as visual-verbal language”.

b Of course, not only do words in language evoke sensory images, the combination of words, like sentences, does that too, but I will not go into this subject as it is not essential for the theory developed here.

c Because sensory-based processes are significantly faster than verbal processes, sensory consciousness will appear with a much shorter delay than the 200 milliseconds usually associated with verbal consciousness.

d That using spoken language requires complex neural processes is not because it provides a complex outcome, but because the transformation of cognitive activity into reasonably usable verbal communication is such a difficult and complex process.

e Einstein is an exceptionally good example of a visual, or rather, sensory thinker, as his intelligence is beyond doubt while his way of thinking and his sometimes laborious use of verbal communication is well documented (39–42]. I will therefore refer to him regularly in this paper.

f A theoretical non-ambiguous language cannot exist for the following reason. Even if a different word were to be assigned to every meaning of any word, for instance by numbering them (meaning1, meaning2 etc.), the definition of these exact meanings would require the use of ambiguous language. Creating a dictionary with the correct text in non-ambiguous language would involve an almost infinite circular process.
